# Selection of reference genes for quantitative real-time PCR analysis in exogenous hormone-treated *Lycoris aurea*: Reference genes selection for *Lycoris aurea*

**DOI:** 10.3724/abbs.2024197

**Published:** 2024-12-04

**Authors:** Wei Zhao, Ying Tian, Yi Wang, Yun Wu, Ziming Ren, Kehu Li

**Affiliations:** 1 Key Laboratory of Plant Resource Conservation and Germplasm Innovation in Mountainous Region (Ministry of Education) Collaborative Innovation Center for Mountain Ecology & Agro-Bioengineering (CICMEAB) Institute of Agro-Bioengineering College of Life Sciences Guizhou University Guiyang 550025 China; 2 College of Horticulture Science Zhejiang A&F University Hangzhou 311300 China; 3Department of Landscape Architecture Zhejiang Sci-Tech University Hangzhou 310018 China

The bulbs of *Lycoris aurea* Herb can produce unique alkaloids, which have been shown to have antiviral, anti-tumor, anti-malarial, and immunostimulatory properties and are effective in the symptomatic treatment of Alzheimer’s disease [1]. As a consequence, the demand for *Lycoris* bulbs has dramatically increased, especially for *L*. *aurea*, which is native to southern China and is highly important for increasing bulb yield [2]. Previous research in this area reported that the use of exogenous hormones is a way to optimize the artificial reproduction of their bulbs [3]. Therefore, studying the molecular mechanism of bulb growth and development is highly important.

Quantitative real-time PCR (qRT-PCR) is widely used in gene expression analysis to explore gene functions that regulate plant growth and development because of its high specificity, accuracy, sensitivity and efficiency, and selecting reference genes correctly is crucial for obtaining proper results and interpretations via qRT-PCR analysis [4]. The selection and validation of reference genes of some *Lycoris* species in different floral development stages and tissues have been carried out, but under some abnormal conditions, including abiotic stress and hormone treatments, have not been assessed [ 5, 6]. In this study, we aimed to identify suitable reference genes for *L*. *aurea* under seven hormone treatments. A total of seven candidate reference genes were selected for investigation. The expressions of these candidate genes were measured by qRT-PCR. The expression stability of candidate genes was comprehensively evaluated by four different statistical algorithms, including geNorm, BestKeeper, NormFinder and RefFinder [ 7– 10].

The bulbs of *L*. *aurea* with similar diameters growing in our greenhouse were selected. For hormone treatments, the bulbs were exposed to 25 mg/L 6-BA (6-benzylaminopurine), 200 mg/L ethrel, 1 mg/L 2,4-EBR (2,4-epi-brassinolide), 100 mg/L MeJA (methyl jasmonate), 100 mg/L GA (gibberellic acid), 0.384 mg/L (1 μM) SL (strigolactone) or 100 mg/L ABA (abscisic acid) for 0, 4, 18, or 48 h. 6-BA (containing NaOH), ethrel, 2,4-EBR (containing absolute ethanol), MeJA (containing absolute ethanol), GA (containing absolute ethanol), SL (containing propanol) and ABA (containing absolute ethanol) were all dissolved in distilled water. Moreover, the bulbs were placed in a plant growth chamber (RXZ-308B; Ningbo Jiangnan Instrument Factoy, Ningbo, China) with a mean temperature of 25±5°C, a relative humidity of 95.0%, and a day/night cycle of 16/8 h. Then, the bulbs were sampled at different time points (0, 4, 18, and 48 h after treatment) for each treatment, with three biological replicates, and snap-frozen in liquid nitrogen. All the samples were subsequently stored at –80°C for subsequent experiments.

Total RNA was extracted from the samples using the MolPure ^®^ Plant RNA kit (Yeasen Biotechnology, Shanghai, China). K5600C (KAIAO Technology Development Co., Ltd., Beijing, China) and 1% agarose gel electrophoresis were used to measure the concentration and quality of the RNA. The first strand of cDNA was synthesized using Hifair ^®^ III 1st Strand cDNA Synthesis SuperMix for qPCR (gDNA digester plus) (Yeasen Biotechnology).

Seven common housekeeping genes, including activating enhancer binding protein 4 ( *AP4*), ubiquitin 4 ( *UBQ4*), β-tubulin ( *TUB2*), eukaryotic translation initiation factor ( *EIF*), ubiquitin conjugating enzyme E2 ( *UBCE*), TATA box binding protein-associated factor ( *TBP*), and UBC core domain-containing protein ( *UBC24*), were selected as candidate reference genes, and all specific primers were designed with Primer Premier 5.0 software ( Table 1). All cDNA samples were mixed equally as a template and diluted in 4 gradients 3 times for each gradient. The specificity of the primers was visualized by 1% agarose gel electrophoresis of the PCR products. The standard curves of each gene were drawn from the results of the qRT-PCR reaction. The amplification efficiency (E) and correlation coefficient (R ^2^) of each primer were calculated.

**Table:** **Table 1** Primers and amplification characteristics of candidate reference genes

Gene name	Gene ID	Primer sequence (5′→3′)	Product (bp)	Tm (°C)	E (%)	R2
*AP4*	Unigene48898_All	F: GACCAACTGGAGAGAGTGCTTGAG	120	61.6	110.1	0.999
R: CGTGTTGCCTGCCTCTATACCTAC	61.3
*UBQ4*	Unigene47709_All	F: TAGTCTTGCGTCTTCGTGGA	198	57.4	96.7	1.000
R: AATCAGCAAGGGTCCTCCCA	57.3
*TUB2*	Unigene7296_All	F: GAGGCGGAGAATTGTGATTGCTTG	150	61.6	98.3	0.997
R: GGACGGGAAGACGGAGAAAGTG	60.0
*EIF*	Unigene95331_All	F: CCTAGTTTGGCTGAGTCTTCTT	116	57.0	108.6	0.993
R: GACCACTGCCGATAGTCATATT	56.0
*UBCE*	Unigene45273_All	F: ATGGCAAGGTGGATGAGAAAGGG	113	59.9	95.4	0.998
R: CATTGACTGCCGTTGAGATGTGAG	61.2
*TBP*	Unigene54738_All	F: CCTAGACCTGGAGAGGTTATTTG	115	56.8	108.9	0.994
R: CTCCCTCTAGTCCTCCTGATATT	57.4
*UBC24*	Unigene96_All	F: GAGGCTGGCTACGACAAACT	127	58.0	95.3	0.999
R: AATGCTTAGGAGGCTTGCGT	58.8

The qRT-PCR experiments of the reference genes were performed with Hieff ^®^ qPCR SYBR Green Master Mix (No Rox) (Yeasen Biotechnology) on the Real-Time system from Bio-Rad Laboratories (Hercules, USA). The reaction system (20 μL) included 10 μL of 1× Hieff ^®^ qPCR SYBR Green Master Mix (No Rox), 2 μL of cDNA, 0.4 μL of each primer (10 μM) and 7.2 μL of ddH _2_O. The reaction program was as follows: 95°C for 5 min, 40 cycles of 95°C for 10 s and 60°C for 30 s, and a melting curve was generated at 60–95°C. Each qRT-PCR was performed in triplicate.

The stability of the seven candidate reference genes was evaluated by four statistical software programs, including geNorm, BestKeeper, NormFinder and RefFinder [ 7– 10]. For the geNorm and NormFinder analyses, according to the formula 2 ^–ΔCt^ (ΔCt = each corresponding Ct value–the minimum Ct value), all of the raw cycle threshold (Ct) values were transformed into relative expression levels, which were then imported into two software programs (geNorm and NormFinder) to evaluate gene expression stability. The stability of reference gene expression within each treatment, as well as across all treatments (all sample subsets), was compared.

**Figure 1:**
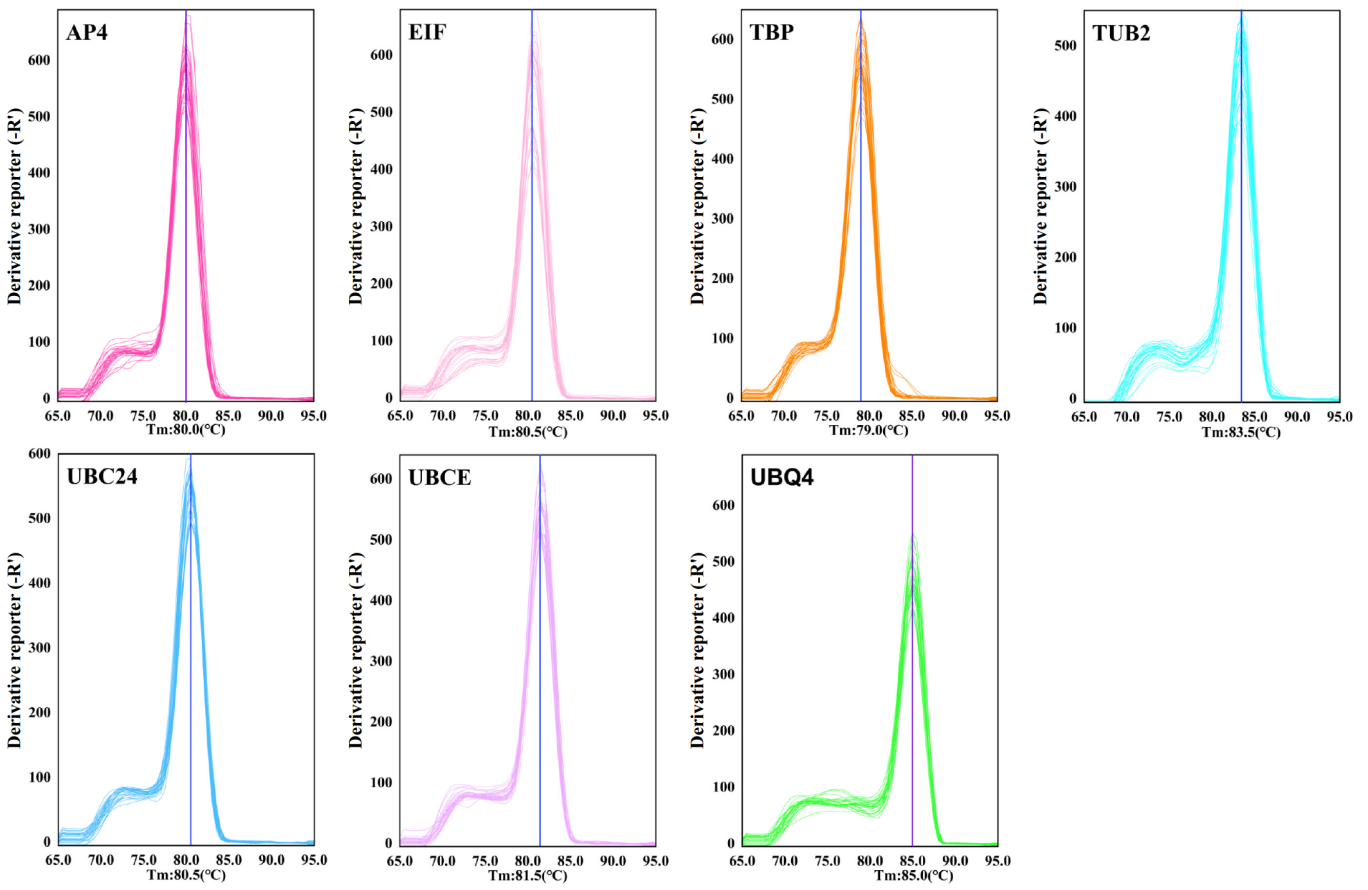
Figure 1 Melting curves of seven candidate reference genes The lines inside the box represent the median values; the lower/upper boundaries of the box indicate the first/third quartiles, respectively. The whiskers represent the minimum and maximum values.

The analysis of the qRT-PCR melting curves revealed that each applied pair of primers generated a single amplification peak ( Figure 1).Furthermore, the amplification efficiencies (E) of all the candidate reference genes ranged from 95.3% to 110.1%, and the linear R ^2^ values (regression coefficients) varied from 0.9993 to 1 ( Table 1). These results indicated the high specificity and suitability of the seven reference genes for qRT-PCR.

**Figure 2:**
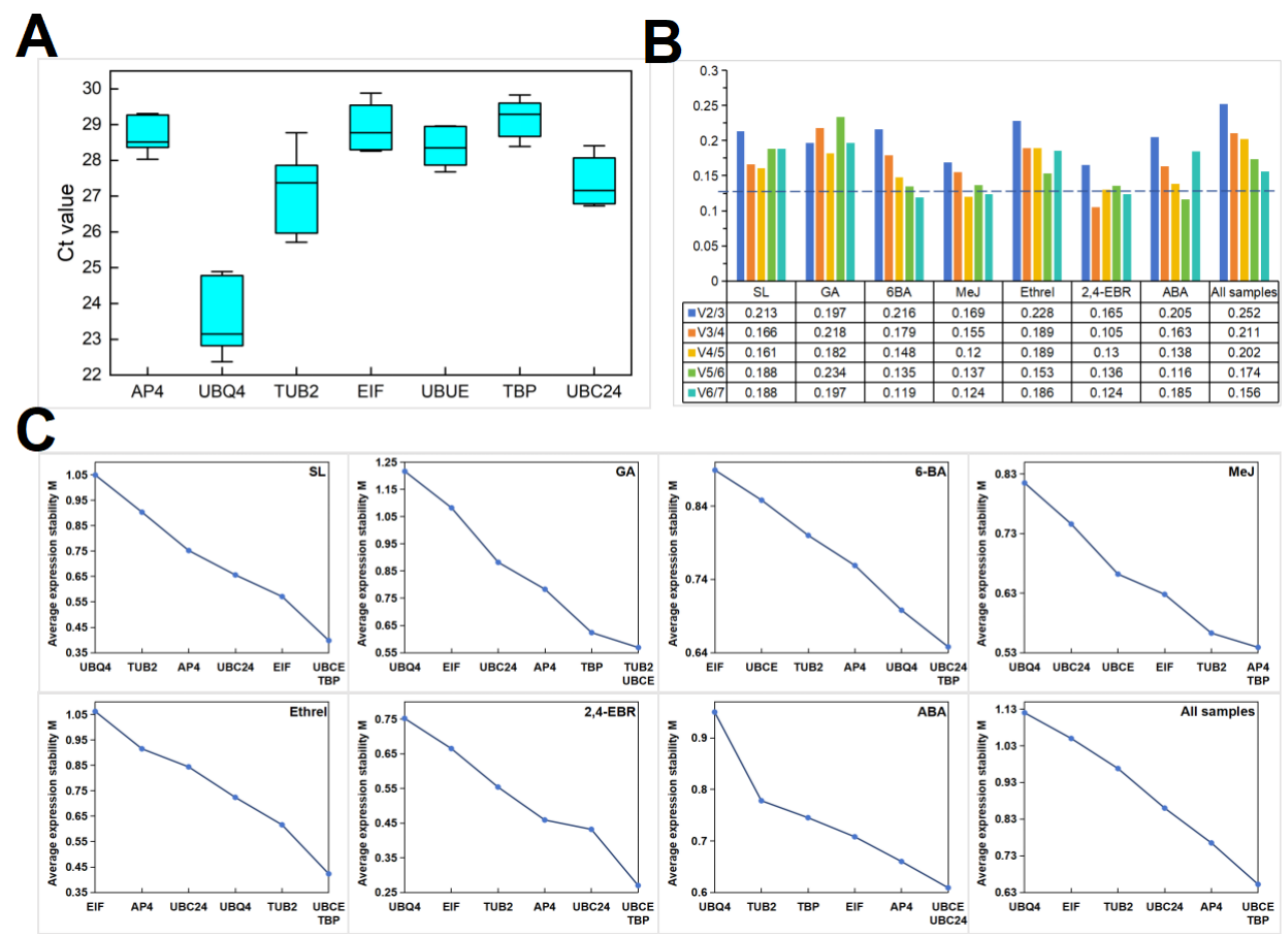
Figure 2 Distribution of Ct values (A), pairwise variation (V _n_/V _n +1_) (B) and average expression stability values (M) (C) of seven candidate reference genes

The Ct refers to the number of amplification cycles at which the fluorescent signal of the amplified product reaches the set fluorescence threshold during qRT-PCR amplification process. A lower Ct value reflects a higher expression level. According to the experimental results, the Ct values of all the candidate reference genes ranged from 22.37 to 29.88. The mean Ct values were mostly between 23.47 and 29.18 across all the tested samples. *UBQ4*, which presented the lowest average Ct value of 23.47 ± 0.98 (mean ± SD), was the most abundantly expressed gene, whereas with a mean Ct value of 29.18 ± 0.54, and *TBP* presented the lowest expression level ( Figure 2A).

For geNorm, the Ct values cannot be imported into the software to assess the best reference genes directly; rather, they need to be transformed to relative expression levels via a formula and then calculated. GeNorm judges the stability of gene expression on the basis of the M value. The more stable the gene expression was, the lower the M value was, but the M value was suggested to be less than 1.5. The M values of all the samples are shown in Figure 2C and Table 2. *UBQ4* was the most unstable gene across the 5 treatments, including the SL (M = 1.049), GA (M = 1.216), MeJ (M = 0.815), 2,4-EBR (M = 0.752), and ABA (M = 0.950) treatments and all the sample subsets (M = 1.120), whereas *EIF* was the worst gene in the other two sets. In the SL, ethrel, and 2,4-EB treatments and all sample subsets, *UBCE* and *TBP* had the lowest M values of 0.398, 0.423, 0.270 and 0.652, respectively. *TUB2* and *UBCE* (M = 0.569) presented the greatest stability in the GA treatment, whereas *UBC24* and *TBP* (M = 0.648) presented the greatest stability in the 6-BA treatment. *AP4* and *TBP* (M = 0.539) were identified as excellent reference genes in the MeJ treatment, whereas *UBCE and UBC24* (M = 0.609) were the best in the ABA treatment. By the geNorm algorithm, the pairwise variation (V _n_/V _n+1_) between the reference genes can also be calculated to determine the appropriate number of reference genes for accurate normalization. The criterion was that if the V _n_/V _n+1_ values were greater than the threshold of 0.15, then the optimal number of reference genes was No. (n+1). As shown in Figure 2B, the pairwise variation (V _2_/V _3_) of all the sets was greater than 1.5, and except the 2,4-EBR treatment, the pairwise variation (V _3_/V _4_) of the other treatments was greater than 1.5. This finding indicates that accurate normalization of tested gene expression can be achieved via three reference genes in the 2,4-EBR; however, the V3/4 values in other sample sets were greater than 0.15, suggesting that more reference genes are needed for accurate normalization of gene expression.

**Table:** **Table 2** The expression stability of the seven candidate reference genes in different hormones treatments calculated by the geNorm, NormFinder, Bestkeeper and RefFinder

Software	Rank	SL	GA	6-BA	MeJ	Ethrel	2,4-EBR	ABA	All samples
geNorm		Gene	M	Gene	M	Gene	M	Gene	M	Gene	M	Gene	M	Gene	M	Gene	M
1	*UBCE*	0.398	*TUB2*	0.569	*UBC24*	0.648	*AP4*	0.539	*UBCE*	0.423	*UBCE*	0.270	*UBCE*	0.609	*UBCE*	0.652
2	*TBP*	0.398	*UBCE*	0.569	*TBP*	0.648	*TBP*	0.539	*TBP*	0.423	*TBP*	0.270	*UBC24*	0.609	*TBP*	0.652
3	*EIF*	0.571	*TBP*	0.624	*UBQ4*	0.698	*TUB2*	0.563	*TUB2*	0.616	*UBC24*	0.432	*AP4*	0.660	*AP4*	0.765
4	*UBC24*	0.656	*AP4*	0.783	*AP4*	0.759	*EIF*	0.628	*UBQ4*	0.724	*AP4*	0.459	*EIF*	0.708	*UBC24*	0.860
5	*AP4*	0.752	*UBC24*	0.882	*TUB2*	0.800	*UBCE*	0.662	*UBC24*	0.844	*TUB2*	0.554	*TBP*	0.745	*TUB2*	0.968
6	*TUB2*	0.903	*EIF*	1.082	*UBCE*	0.848	*UBC24*	0.746	*AP4*	0.915	*EIF*	0.665	*TUB2*	0.778	*EIF*	1.050
7	*UBQ4*	1.049	*UBQ4*	1.216	*EIF*	0.889	*UBQ4*	0.815	*EIF*	1.062	*UBQ4*	0.752	*UBQ4*	0.950	*UBQ4*	1.120

NormFinder		Gene	SV	Gene	SV	Gene	SV	Gene	SV	Gene	SV	Gene	SV	Gene	SV	Gene	SV
1	*TBP*	0.356	*UBCE*	0.191	*TUB2*	0.422	*AP4*	0.203	*UBCE*	0.227	*UBCE*	0.135	*AP4*	0.518	*UBCE*	0.220
2	*UBCE*	0.387	*TUB2*	0.373	*UBC24*	0.490	*TBP*	0.372	*TBP*	0.326	*TBP*	0.326	*UBC24*	0.519	*TBP*	0.392
3	*UBC24*	0.639	*TBP*	0.629	*UBQ4*	0.630	*TUB2*	0.445	*TUB2*	0.608	*TUB2*	0.460	*EIF*	0.522	*UBC24*	0.647
4	*UBQ4*	0.660	*AP4*	0.888	*TBP*	0.659	*EIF*	0.600	*UBC24*	0.769	*UBC24*	0.504	*UBCE*	0.550	*EIF*	0.899
5	*EIF*	0.683	*UBC24*	1.001	*UBCE*	0.682	*UBCE*	0.647	*UBQ4*	0.772	*AP4*	0.586	*TUB2*	0.612	*TUB2*	1.493
6	*AP4*	0.835	*EIF*	1.231	*EIF*	0.814	*UBC24*	0.739	*AP4*	0.829	*EIF*	0.756	*TBP*	0.666	*AP4*	1.908
7	*TUB2*	0.915	*UBQ4*	1.368	*AP4*	1.085	*UBQ4*	0.882	*EIF*	1.457	*UBQ4*	0.860	*UBQ4*	1.221	*UBQ4*	2.500

Bestkeeper		Gene	SD	CV	Gene	SD	CV	Gene	SD	CV	Gene	SD	CV	Gene	SD	CV	Gene	SD	CV	Gene	SD	CV	Gene	SD	CV
1	*AP4*	0.716	2.556	*UBCE*	0.437	1.580	*TUB2*	0.590	2.588	*UBQ4*	0.745	3.329	*UBCE*	0.923	3.230	*AP4*	1.111	3.817	*TBP*	1.031	3.454	*TBP*	0.727	2.493
2	*UBC24*	0.815	2.922	*TUB2*	0.489	1.901	*UBC24*	0.630	2.356	*AP4*	0.917	3.230	*TBP*	1.030	3.482	*UBQ4*	1.276	5.151	*EIF*	1.115	3.732	*UBCE*	0.765	2.700
3	*UBQ4*	0.856	3.696	*TBP*	0.563	1.982	*TBP*	0.688	2.349	*TUB2*	0.953	3.535	*AP4*	1.086	3.710	*UBC24*	1.299	4.629	*AP4*	1.129	3.851	*UBC24*	0.884	3.219
4	*UBCE*	1.064	3.754	*AP4*	0.811	2.862	*UBQ4*	0.779	2.734	*TBP*	1.053	3.673	*UBC24*	1.160	4.272	*TUB2*	1.339	4.807	*UBCE*	1.337	4.617	*EIF*	1.030	3.551
5	*TBP*	1.085	3.758	*EIF*	0.893	3.024	*UBCE*	0.795	2.851	*UBC24*	1.114	4.157	*TUB2*	1.165	4.233	*EIF*	1.348	4.576	*TUB2*	1.340	4.656	*AP4*	1.531	5.479
6	*TUB2*	1.153	4.440	*UBC24*	1.077	3.973	*AP4*	0.856	3.696	*UBCE*	1.180	4.204	*UBQ4*	1.286	5.584	*UBCE*	1.349	4.660	*UBC24*	1.344	4.731	*TUB2*	1.599	6.030
7	*EIF*	1.163	4.109	*UBQ4*	1.171	5.033	*EIF*	0.996	3.463	*EIF*	1.324	4.687	*EIF*	1.739	6.049	*TBP*	1.370	4.630	*UBQ4*	1.635	6.570	*UBQ4*	1.655	6.816

RefFinder		Gene	RV	Gene	RV	Gene	RV	Gene	RV	Gene	RV	Gene	RV	Gene	RV	Gene	RV
1	*UBCE*	2.450	*UBCE*	1.570	*TUB2*	1.860	*AP4*	1.320	*UBCE*	1.190	*UBCE*	1.630	*AP4*	1.320	*UBCE*	1.570
2	*TBP*	2.550	*TUB2*	2.280	*UBC24*	2.110	*TBP*	3.410	*TBP*	2.000	*TBP*	2.380	*TBP*	2.740	*TBP*	1.570
3	*UBC24*	4.160	*TBP*	4.230	*UBQ4*	5.690	*TUB2*	4.000	*TUB2*	3.830	*AP4*	3.660	*EIF*	2.910	*UBC24*	5.000
4	*AP4*	4.880	*AP4*	6.240	*TBP*	5.730	*UBQ4*	5.200	*UBQ4*	6.240	*UBC24*	3.940	*UBC24*	3.740	*EIF*	6.480
5	*UBQ4*	5.630	*UBC24*	7.970	*UBCE*	7.480	*UBCE*	6.450	*UBC24*	6.960	*TUB2*	4.950	UBCE	4.470	*TUB2*	8.240
6	*EIF*	6.770	*EIF*	8.740	*EIF*	9.240	*EIF*	6.470	*AP4*	7.330	*UBQ4*	6.690	TUB2	6.000	*AP4*	8.740
7	*TUB2*	9.740	*UBQ4*	10.000	*AP4*	9.740	*UBC24*	7.440	*EIF*	10.000	*EIF*	8.130	UBQ4	10.000	*UBQ4*	10.000

The NormFinder algorithm was used to analyze the expression stability of the candidate genes further. All SV values were calculated via the NormFinder program ( Table 2). According to the SV values, *TBP* (0.356) was the most stable gene in the SL subset, whereas *TUB2* (0.915) was the least stable gene. Among the subset of 6-BA, *TUB2* (0.422) presented the highest stability, whereas *AP4* (1.085) was the most unstable gene. *AP4* (0.203/0.518) was the best in the subset of MeJ and ABA, whereas *UBQ4* (0.882/1.221) was the worst. In addition, *UBCE* (0.191/0.227/0.135/0.220) was the most stably expressed gene in the sets of GA, Ethrel, 2,4-EBR and all samples subset, whereas *UBQ4* and *EIF* were the most unstable genes in the five sets.

BestKeeper is also a program for analyzing the stability of gene expression by comparing the values of standard deviation (SD) and coefficient of variation (CV). Generally, the more stable the gene expression is, the lower the SD and CV values. The stability ranking results of the seven candidate reference genes are shown in Table 2. AP4 was the most stably expressed gene under the SL and 2,4-EBR treatments. *TUB2* and *UBQ4* were the appropriate reference genes for 6-BA and MeJ, respectively. In particular, *UBCE* expression showed the highest stability in the GA set and ethrel. Among the sets of ABA genes and all sample subsets, *TBP* was the best gene. In view of the ranking results, *UBQ4* and *TBP* were the most unstable genes for GA and 2,4-EBR. *UBQ4* was ranked last under ABA treatment and in all sample subsets. In addition, *EIF* was the gene with the most unstable expression in all the other sets.

Because of the differences in the results that the algorithms of geNorm, NormFinder and BestKeeper may generate, the results obtained using the three software programs might be inconsistent. Hence, a comprehensive ranking of the stability of reference genes was required to be roundly evaluated and obtained by RefFinder. *UBCE* was the most suitable reference gene for normalization under the SL, GA, ethrel, and 2,4-EBR treatments and for all sample subsets, whereas for MeJ and ABA, *AP4* was the most stable reference gene ( Table 2). Additionally, comprehensive ranking calculations revealed that *TUB2* was the most stable gene under the 6-BA treatment. In particular, *UBQ4* was the least stable reference gene across the three treatments (GA, ABA and all sample subsets). *TUB2* and *AP4* were the most unstable reference genes in the SL set and the 6-BA treatment, respectively. In the set of MeJ genes, *UBC24* was the most unstable reference gene. In addition, *UBCE* ranked among the three most stable genes in the five sets, and it can be regarded as a relatively stable reference gene in the bulbs of *L*. *aurea*.

For quantifying gene expression, selecting suitable reference genes is an important prerequisite by qRT-PCR. Here, we verified a set of candidate reference genes for the normalization of gene expression using qRT-PCR in *L*. *aurea* under different hormone treatments. The expression stability of the seven candidates was evaluated via four common professional programs, namely, geNorm, NormFinder, BestKeeper and RefFinder. For the study of gene expression under SL, ethrel, and 2,4-EBR treatments and all sample subsets, we recommend *UBCE* and *TBP* for normalization of the qRT-PCR data. For gene expression studies under GA treatment, *UBCE* and *TUB2* were the two most suitable reference genes. In addition, we identified the stable reference genes *TUB2* and *UBC24* for the 6-BA treatment and *AP4* and *TBP* for the MeJ treatment. These results will contribute to further quantitative analysis of gene expression related to exogenous hormone treatment in *L*. *aurea* and indicate that it is important to identify specific reference genes for specific conditions in *L*. *aurea*.
